# Serum DSG2 as a potential biomarker for diagnosis of esophageal squamous cell carcinoma and esophagogastric junction adenocarcinoma

**DOI:** 10.1042/BSR20212612

**Published:** 2022-05-06

**Authors:** Yin-Qiao Liu, Ling-Yu Chu, Tian Yang, Biao Zhang, Zheng-Tan Zheng, Jian-Jun Xie, Yi-Wei Xu, Wang-Kai Fang

**Affiliations:** 1 Department of Biochemistry and Molecular Biology, Shantou University Medical College, Shantou, China; 2 Department of Gastrointestinal Surgery, The First Affiliated Hospital of Shantou University Medical College, Shantou, China; 3 Department of Clinical Laboratory Medicine, The Cancer Hospital of Shantou University Medical College, Shantou, China

**Keywords:** diagnosis, DSG2, esophageal squamous cell carcinoma, esophagogastric junction adenocarcinoma, serum biomarker

## Abstract

**Background**: Exploration of serum biomarkers for early detection of upper gastrointestinal cancer is required. Here, we aimed to evaluate the diagnostic potential of serum desmoglein-2 (DSG2) in patients with esophageal squamous cell carcinoma (ESCC) and esophagogastric junction adenocarcinoma (EJA).

**Methods**: Serum DSG2 levels were measured by enzyme-linked immunosorbent assay (ELISA) in 459 participants including 151 patients with ESCC, 96 with EJA, and 212 healthy controls. Receiver operating characteristic (ROC) curves were used to evaluate diagnostic accuracy.

**Results**: Levels of serum DSG2 were significantly higher in patients with ESCC and EJA than those in healthy controls (*P*<0.001). Detection of serum DSG2 demonstrated an area under the ROC curve (AUC) value of 0.724, sensitivity of 38.1%, and specificity of 84.8% for the diagnosis of ESCC in the training cohort, and AUC 0.736, sensitivity 58.2%, and specificity 84.7% in the validation cohort. For diagnosis of EJA, measurement of DSG2 provided a sensitivity of 29.2%, a specificity of 90.2%, and AUC of 0.698. Similar results were observed for the diagnosis of early-stage ESCC (AUC 0.715 and 0.722, sensitivity 36.3 and 50%, and specificity 84.8 and 84.7%, for training and validation cohorts, respectively) and early-stage EJA (AUC 0.704, sensitivity 44.4%, and specificity 86.9%). Analysis of clinical data indicated that DSG2 levels were significantly associated with patient age and histological grade in ESCC (*P*<0.05).

**Conclusion**: Serum DSG2 may be a diagnostic biomarker for ESCC and EJA.

## Introduction

Esophageal cancer ranks seventh in terms of incidence and sixth for cancer-related deaths worldwide [1], and is among the most invasive and metastatic malignancies, representing a serious global health problem [2]. There are two main histologic types of esophageal cancer: esophageal squamous cell carcinoma (ESCC) and esophageal adenocarcinoma (EAC) [3]. ESCC is the most prevalent form of esophageal cancer, accounting for 70% of cases. Notably, the prevalence of ESCC has decreased in recent years, while the incidence of esophagogastric junction adenocarcinoma (EJA) is increasing significantly worldwide [4–6]. In China, EJA is also prevalent in areas with high incidence rates of ESCC [7]. Smoking and alcohol consumption account for more than 90% of ESCC in the Western world [8], but are not important contributing factors in ESCC occurrence in China [7]. Nevertheless, geographical differences in incidence rates in China strongly suggest that there are major etiological environmental or lifestyle factors influencing the development of ESCC and EJA [9].

Patients with ESCC have a poor prognosis, with a 5-year survival rate of less than 20% [10]. The high mortality of ESCC and EJA is mainly due to advanced stage at diagnosis and a lack of early specific biomarkers [11]. Early detection and treatment of esophageal lesions can significantly improve prognosis and reduce mortality [12]; however, there remains a lack of effective strategies to detect precancerous lesions and early ESCC and EJA [13]. Although endoscopy can be used as a primary screening technique to identify ESCC and EJA at an early stage, it is a traumatic procedure with high cost and potentially significant side effects, and its widespread use is limited [14,15]. Therefore, the discovery of tumor serum biomarkers is crucial for the diagnosis and treatment of ESCC and EJA.

Desmoglein-2 (DSG2) is a transmembrane glycoprotein belonging to the desmosomal cadherin family that plays an important role in desmosome junctions, forming cell–cell junctions, and acting as an anchor for intermediate filaments [16]. DSG2 not only mediates intercellular adhesion, but also acts as a signaling scaffold for cell movement [17]. Abnormally high expression of DSG2 is closely associated with poor prognosis in multiple types of cancer, including skin cancer [18], colon cancer [19], non-small cell lung cancer [20], lung adenocarcinoma [21,22], stomach cancer [23], breast cancer [24], and hepatocellular cancer [25], making it an appealing candidate serum biomarker for certain tumors. Further, studies using enzyme-linked immunosorbent assay (ELISA) have confirmed that the high expression of DSG2 in serum from patients with head and neck squamous cell carcinoma (HNSCC) can serve as a potential biomarker [26]. In addition, Fang et al. indicated that DSG2 was significantly overexpressed in ESCC [27]; however, serum DSG2 has not been demonstrated as a clinical biomarker in patients with ESCC and EJA. Therefore, in the present study, we aimed to evaluate the expression of DSG2 in serum from patients with ESCC and EJA and whether it has potential for use as a diagnostic biomarker.

## Materials and methods

### Study participants

In the present study, 302 serum samples, including 151 from patients with ESCC and 151 from healthy controls, were collected from the Cancer Hospital of Shantou University Medical College and Cancer Centre of Sun Yat-sen University (SYSU), from June 2018 to September 2020. Serum samples from 96 patients with EJA and 61 healthy controls were collected from The First Affiliated Hospital of Shantou University Medical College, from January 2018 to November 2018. Healthy controls were qualified blood donors, none of whom had evidence of cancer, and were recruited from the Physical Examination Center at the same hospital. Cases in the cancer group were all newly diagnosed patients who had not received any anticancer treatment before blood collection, and whose follow-up data were complete. Serum samples were coagulated at room temperature for 30 min before centrifugation at 1250×***g*** for 5 min and then stored at −80°C until the experiment started.

The diagnosis of ESCC and EJA was confirmed histopathologically, and tumor staging was consistent with the eighth edition of the American Joint Committee on Cancer (AJCC) Cancer Staging Manual [28]. AJCC TNM stage 0+I+IIA was defined as early stage, as in our previous study [29].

### ELISA for DSG2

ELISA kits to assess serum DSG2 level were purchased from RayBio® (Catalog number: ELH-DSG2.; U.S.A.), according to the user manual. Briefly, reagents and samples were prepared as instructed, serum samples diluted to 1:1, and the standard was diluted to concentrations of 4000, 1600, 640, 256, 102.4, 40.96, 16.38, and 0 pg/ml. Then, 100 μl of each standard and sample were added to appropriate wells and incubated at room temperature for 2.5 h. Plates were washed four-times using a microplate washer (Thermo Fisher Scientific) and 100 μl Biotin-antibody (1×) added to each well and incubated at room temperature for 1 h, followed by a further four washes using the microplate washer. Prepared streptavidin solution (1×, 100 μl) was added to each well and the plates incubated at room temperature for 45 min. After washing, 100 μl of TMB One-Step Substrate Reagent was added to each well and then incubated at room temperature for 30 min. Finally, 50 μl of stop solution was added to terminate the reaction, and optical density (OD) values read at wavelengths of 450 and 590 nm using a plate microplate reader (Thermo Fisher Scientific). OD values were converted into concentration using the standard curve and then multiplied by the dilution factor. Two replicates of each serum sample were analyzed and mean values calculated.

### Statistical analysis

Data analyses were conducted using Sigma Plot (version 10.0), GraphPad Prism (version 7.0), Microsoft Excel, and SPSS for Windows (version 19.0). The significance of differences in serum DSG2 levels between groups were tested using the Mann–Whitney U test. Sensitivity, specificity, and areas under the curve (AUCs) values with 95% confidence interval (CI) were determined by plotting receiver operating characteristic (ROC) curves. Optimum cut-off values were obtained from Youden’s index values of the ROC curves, which yield maximum values of sensitivity plus (100%—specificity). Positive predictive value (PPV), negative predictive value (NPV), false positive rate (FPR), false negative rate (FNR), positive likelihood ratio (PLR), and negative likelihood ratio (NLR) were obtained using the optimal cut-off values. Associations between clinical characteristics and levels of DSG2 were evaluated using the chi-square test. In all statistical tests, *P*-values (two sided) <0.05 were considered statistically significant.

## Results

### Serum DSG2 levels in patients with ESCC and EJA and healthy controls

**Figure 1:**
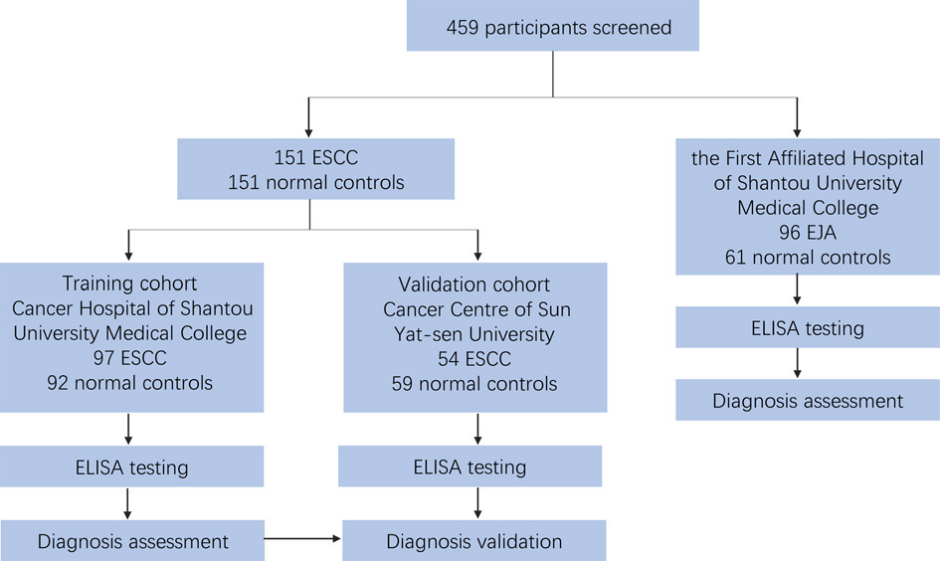
Study flow chart

To evaluate the level of DSG2 in serum samples, 459 participants were selected, including 97 patients with ESCC and 92 healthy volunteers in the training cohort; 54 patients with ESCC and 59 healthy volunteers in the validation cohort; and 96 patients with EJA and 61 healthy volunteers (Figure 1). The clinical features of patients and healthy controls are summarized in Table 1.

**Table 1:** Participant information and clinicopathological characteristics

Group	Training cohort		Validation cohort		EJA (*n*=96)	Healthy control (*n*=61)
	ESCC (*n*=97)	Healthy control (*n=*92)	ESCC (*n*=54)	Healthy control (*n*=59)		
Age, years						
Mean ± SD	63 ± 8	51 ± 8	61 ± 9	52 ± 9	64 ± 10	48 ± 12
Range	46–83	33–77	44–81	40–80	22–81	29–81
Sex						
Male	72	57	49	35	77	41
Female	25	35	5	24	19	20
Smoker						
Yes	64		39		27	
No	33		15		65	
Unknown	0		0		4	
Alcohol consumption						
Yes	37		15		10	
No	60		39		38	
Unknown	0		0		48	
Site of tumor						
Upper	11		0		NA	
Middle	64		33			
Lower	11		18			
Unknown	11		3			
Size of tumor (cm)					NA	
<3	21		23			
≥3	36		22			
Unknown	40		9			
Histological grade						
High	10		2		NA	
Middle	14		22			
Low	20		11			
Unknown	53		19			
Depth of tumor invasion						
Tis	0		2		1	
T1	3		6		3	
T2	8		3		5	
T3	21		33		12	
T4	42		3		48	
Unknown	23		7		27	
Lymph node metastasis						
N0	20		25		23	
N1	33		9		13	
N2	18		9		14	
N3	9		5		18	
Unknown	17		6		28	
TNM stage						
0	0		1		0	
I	6		5		6	
II	10		18		8	
III	35		18		45	
IV	36		5		24	
Unknown	10		7		13	

**Figure 2:**
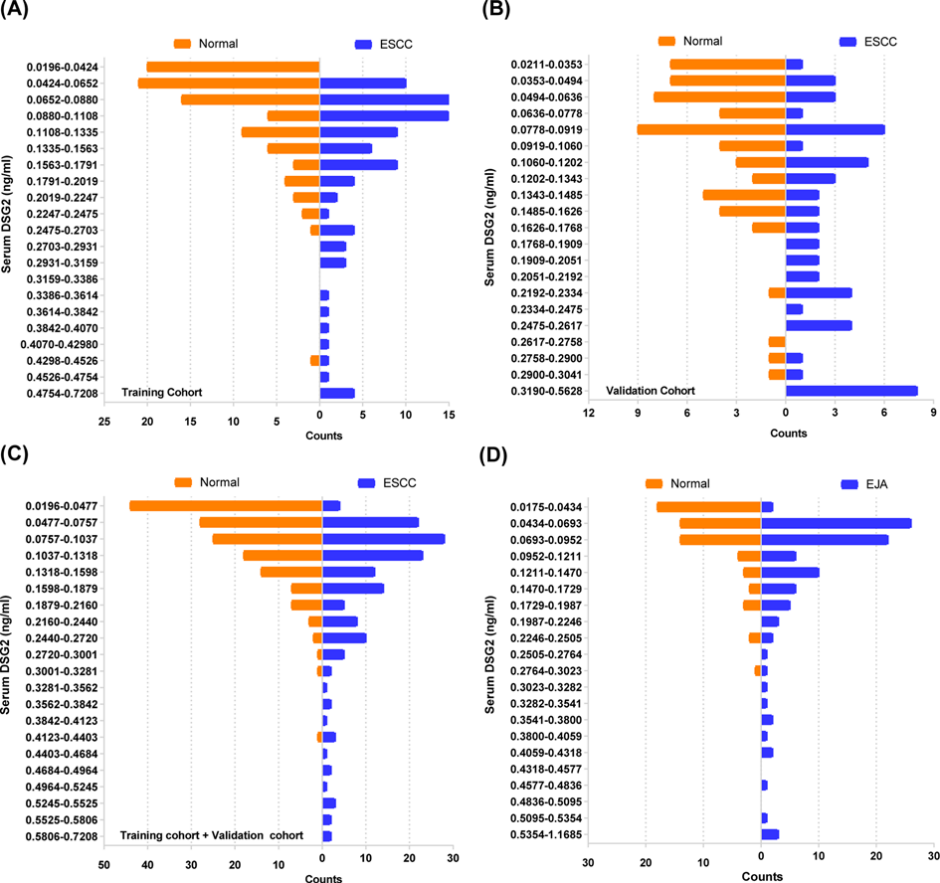
Frequency distribution of DSG2 levels in serum from patients with ESCC and EJA, and healthy controls (**A**) In healthy controls in the ESCC training cohort, the lowest concentration of DSG2 was 0.0196 ng/ml and the highest was 0.7208 ng/ml. (**B**) In healthy controls in the ESCC validation cohort, the lowest DSG2 concentration was 0.0211 ng/ml and the highest was 0.5628 ng/ml. (**C**) In healthy controls in the ESCC joint cohort, the lowest DSG2 concentration was 0.0196 ng/ml and the highest was 0.7208 ng/ml. (**D**) In healthy controls in the EJA cohort, the lowest DSG2 concentration was 0.0175 ng/ml and the highest was 1.1685 ng/ml. Concentrations were divided into 20 equal sections, those with higher concentrations in patients with ESCC and EJA were merged, because no samples was more than those in normal controls. Patients with ESCC and EJA accounted for greater histogram volume at higher concentrations, while more samples from the healthy control groups had lower concentrations of DSG2.

**Figure 3:**
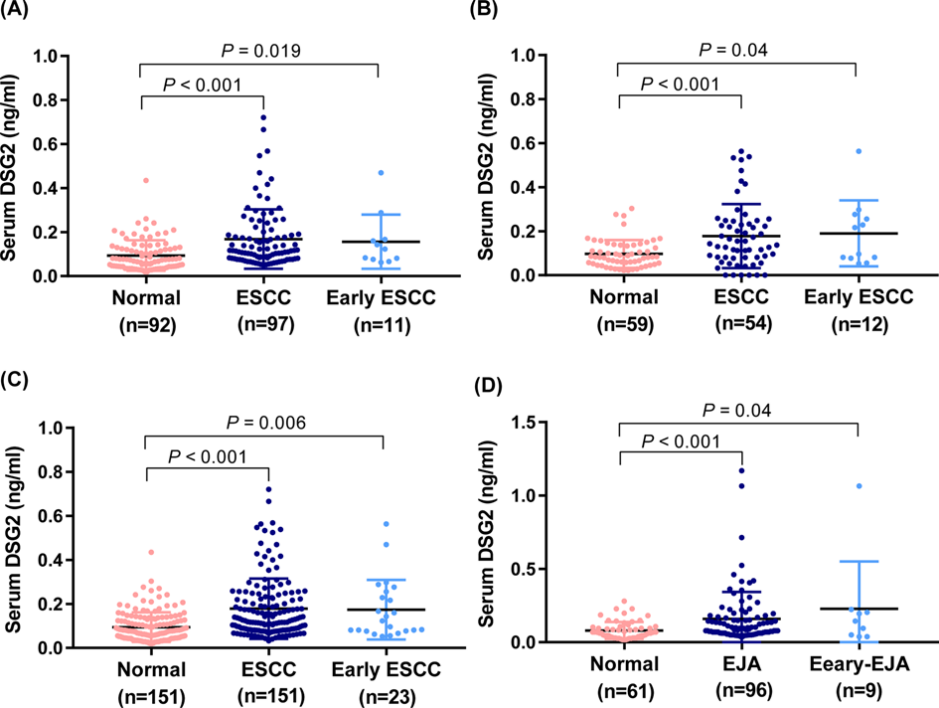
Levels of serum DSG2 (**A**) Scatter plots of serum DSG2 concentrations in healthy controls, and patients with ESCC and early-stage ESCC in the training cohort. (**B**) Scatter plots of serum DSG2 concentrations in healthy controls, and patients with ESCC and early-stage ESCC in the validation cohort. (**C**) Scatter plots of serum DSG2 concentrations from healthy controls, and patients with ESCC and early-stage ESCC in the combined cohort. (**D**) Scatter plots of serum DSG2 concentrations in healthy controls, and patients with EJA and early-stage EJA patients. Black horizontal lines represent mean and error bars standard error values.

Initial analysis of serum DSG2 levels indicated a difference in distributions between the healthy control and patient groups, where the patient group accounted for more histogram volume at higher DSG2 concentrations and the healthy control group accounted for more histogram volume at lower concentrations (Figure 2). In patients with ESCC in the training cohort, mean ± SD serum DSG2 concentration was 0.168 ± 0.135 ng/ml, while values in the early-stage disease and healthy control groups were 0.156 ± 0.123 and 0.093 ± 0.069 ng/ml, respectively (Table 2). To better visualize the distribution and degree of dispersion, scatter plots of serum DSG2 levels in each group were generated (Figure 3). Patients with ESCC had significantly higher serum DSG2 levels than healthy controls (*P*<0.001) (Figure 3). As shown in Figure 3 and Table 2, the difference between patients with early-stage ESCC and healthy controls was also significant (*P*=0.019). In the validation and combined cohorts, serum DSG2 levels were also higher in patients with ESCC than controls (Figure 3 and Table 2). Similar results were observed in EJA (Figure 3 and Table 2).

**Table 2:** Comparison of DSG2 levels among the eight study groups

Group	N	Serum DSG2 (ng/ml)	*P*-value*
		Mean ± SD	
Training cohort			
ESCC	97	0.168 ± 0.135	<0.001
Early-stage ESCC	11	0.156 ± 0.123	0.019
Healthy controls	92	0.093 ± 0.069	
Validation cohort			
ESCC	54	0.198 ± 0.140	<0.001
Early-stage ESCC	12	0.190 ± 0.150	0.04
Healthy controls	59	0.097 ± 0.063	
Training cohort + Validation cohort			
ESCC	151	0.179 ± 0.137	<0.001
Early-stage ESCC	23	0.174 ± 0.136	0.006
Healthy controls	151	0.095 ± 0.067	
EJA	96	0.159 ± 0.184	<0.001
Early-stage EJA	9	0.228 ± 0.322	0.04
Healthy controls	61	0.080 ± 0.058	

*Compared with healthy controls.

### The diagnostic value of DSG2 in ESCC and EJA

**Figure 4:**
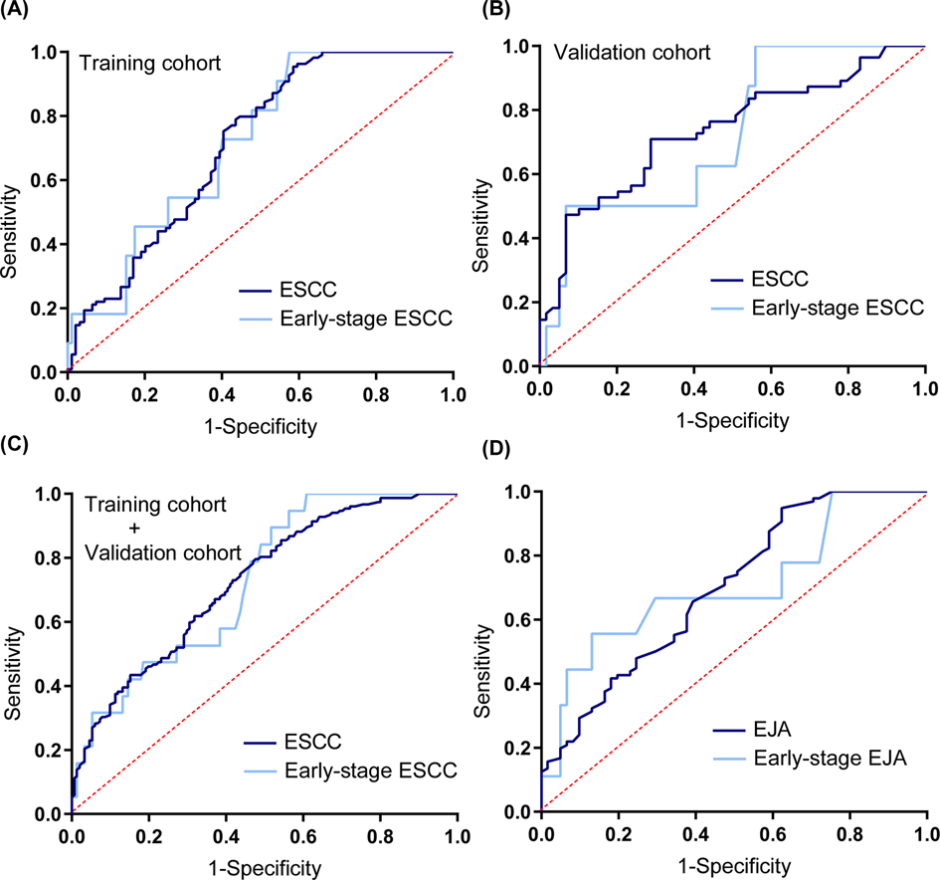
ROC curve analysis of serum DSG2 for the diagnosis of ESCC and EJA (**A**) ROC curve of serum DSG2 for patients with ESCC and early-stage ESCC versus healthy controls in the training cohort. (**B**) ROC curve of serum DSG2 for patients with ESCC and early-stage ESCC versus healthy controls in the validation cohort. (**C**) ROC curve of serum DSG2 for patients with ESCC and early-stage ESCC versus healthy controls in the joint cohort. (**D**) ROC curve of serum DSG2 for patients with EJA and early-stage EJA versus healthy controls.

ROC curves were used to assess the value of serum DSG2 for diagnosis of ESCC and EJA. According to ROC curve analysis, the optimum cut-off value for both ESCC and EJA was 0.150 ng/ml. Analysis of all patients with ESCC in the training cohort indicated that DSG2 had an AUC value of 0.724 (95% CI: 0.652–0.796) for distinguishing individuals with ESCC from healthy controls, with sensitivity/specificity of 38.1% (95% CI: 28.6–48.6%)/84.8% (95% CI: 75.4–91.1%) (Figure 4 and Table 3). DSG2 could also identified early-stage ESCC with a similar AUC value of 0.715 (95% CI: 0.584–0.847), a sensitivity of 36.3% (95% CI: 12.4–68.4%), and a specificity of 84.8% (95% CI: 75.4–91.1%). In the validation and joint cohorts, we found similar diagnostic performance to those determined using training cohort data when the same cut-off value was used (Table 3). When we analyzed the diagnostic values of serum DSG2 in EJA separately, the AUC value for EJA was 0.698 (95% CI: 0.613–0.783). Using a cut-off value of 0.150 ng/ml, DSG2 had a sensitivity of 29.2% (95% CI: 20.6–39.5%) and specificity of 90.2% (95% CI: 79.1–96%) in patients with EJA. Early-stage EJA had an AUC value of 0.704, sensitivity 44.4% (95% CI: 15.3–77.3%), and specificity 86.9% (95% CI: 75.2–93.8%) (Table 3). To improve clinical interpretation, we also present predictive values and likelihood ratios for use of DSG2 for the diagnosis of ESCC and EJA (Table 3).

**Table 3:** Evaluation of serum DSG2 as a diagnostic marker for ESCC

	AUC (95% CI)	Sensitivity	Specificity	FPR	FNR	PPV	NPV	PLR	NLR
ESCC vs. HC									
All stages									
Training cohort	0.724 (0.652–0.796)	38.1%	84.8%	27.5%	43.5%	72.5%	56.5%	2.51	0.73
Validation cohort	0.736 (0.646–0.827)	58.2%	84.7%	22.0%	31.5%	78.0%	68.5%	3.81	0.49
Training + Validation	0.731 (0.676–0.787)	43.7%	84.8%	25.8%	39.9%	74.2%	60.1%	2.87	0.66
Early-stage									
Training cohort	0.715 (0.584–0.847)	36.3%	84.8%	77.8%	8.2%	22.2%	91.8%	2.39	0.75
Validation cohort	0.688 (0.512–0.863)	50.0%	84.7%	60.0%	10.7%	40.0%	89.3%	3.28	0.59
Training + Validation	0.713 (0.607–0.819)	43.5%	84.8%	69.7%	9.2%	30.0%	90.8%	2.85	0.67
EJA vs. HC									
All stages	0.698 (0.613–0.783)	29.2%	90.2%	17.6%	55.3%	82.4%	44.7%	2.97	0.79
Early-stage	0.704 (0.501–0.907)	44.4%	86.9%	66.7%	8.6%	33.3%	91.4%	3.39	0.64

Abbreviation: HC, healthy control.

### Associations between serum DSG2 concentration and clinicopathological features

The data presented in Tables 4 and 5 demonstrate the relationships between levels of serum DSG2 and clinicopathological features of ESCC and EJA, respectively. Levels of DSG2 in the joint ESCC cohort were significantly associated with patient age and histological grade (*P*<0.05), but not with other analyzed factors, including sex, smoking, depth of invasion, metastasis, and TNM stage (Table 4). Further, levels of DSG2 were not significantly associated with any clinical features in the ESCC training cohort, ESCC validation cohort (Table 4), or EJA (Table 5).

**Table 4:** Associations between serum DSG2 level and clinical factors in patients with ESCC

Variable	Training cohort			Validation cohort			Training + Validation		
	*n*	Positive	*P*	*n*	Positive	*P*	*n*	Positive	*P*
Patient age (years)									
<60	32	17 (53.1%)	0.033	22	13 (59.1%)	0.510	54	30 (55.6%)	0.029
≥60	65	20 (30.8%)		32	16 (50.0%)		97	36 (37.1%)	
Sex									
Male	72	29 (40.3%)	0.463	49	27 (55.1%)	0.519	121	56 (46.3%)	0.201
Female	25	8 (32.0%)		5	2 (40.0%)		30	10 (33.3%)	
Tobacco use									
Yes	64	27 (42.26%)	0.254	39	19 (48.7%)	0.236	103	46 (44.7%)	0.730
No	33	10 (30.3%)		15	10 (66.7%)		48	20 (41.7%)	
Alcohol use									
Yes	37	16 (43.2%)	0.417	15	10 (66.7%)	0.236	52	26 (50.0%)	0.259
No	60	21 (35.0%)		39	19 (38.8%)		99	40 (40.4%)	
Site of tumor									
Upper	11	5 (45.5%)	0.84	0	0 (0.0%)	0.618	11	5 (45.5%)	0.919
Middle	64	25 (39.1%)		33	17 (51.5%)		97	42 (43.3%)	
Lower	11	3 (27.3%)		18	11 (61.1%)		29	14 (48.3%)	
Unknown	11	4 (36.4%)		3	1 (33.3%)		14	5 (35.7%)	
Size of tumor (cm)									
<3	21	9 (42.9%)	0.258	23	13 (56.5%)	0.526	44	22 (50.0%)	0.426
≥3	36	16 (44.4%)		22	10 (45.5%)		58	26 (44.8%)	
Unknown	40	12 (30.0%)		9	6 (66.7%)		49	18 (24.5%)	
Histological grade									
High	10	6 (60.0%)	0.417	2	1 (50.0%)	0.134	12	7 (58.3%)	0.041
Middle	14	6 (42.9%)		22	16 (72.7%)		36	22 (61.1%)	
Low	20	6 (30.0%)		11	4 (36.4%)		31	10 (32.3%)	
Unknown	53	19 (35.8%)		19	8 (42.1%)		72	27 (37.5%)	
Depth of tumor invasion									
T1	3	1 (33.3%)	0.719	6	5 (83.3%)	0.496	9	6 (66.7%)	0.547
T2	8	2 (25%)		3	1 (33.3%)		11	3 (27.3%)	
T3	21	10 (47.6%)		33	16 (48.5%)		54	26 (48.1%)	
T4	42	17 (40.5%)		3	1 (33.3%)		45	18 (40.4%)	
Unknown	23	7 (30.4%)		7	5 (71.4%)		30	12 (40.0%)	
Lymph node metastasis									
N0	20	8 (40.0%)	0.898	25	12 (48.0%)	0.375	45	20 (44.4%)	0.854
N1	33	12 (36.4%)		9	6 (66.7%)		42	18 (42.9%)	
N2	18	8 (44.4%)		9	3 (33.3%)		27	11 (40.7%)	
N3	9	4 (44.4%)		5	4 (80.0%)		14	8 (57.1%)	
Unknown	17	5 (29.4%)		6	4 (66.7%)		23	9 (39.1%)	
TNM stage									
I	6	1 (16.7%)	0.47	5	4 (80.0%)	0.428	11	5 (45.5%)	0.895
II	10	6 (60.0%)		18	7 (38.9%)		28	13 (46.4%)	
III	35	13 (37.1%)		18	9 (50.0%)		53	22 (41.5%)	
IV	36	14 (40.0%)		5	3 (60.0%)		41	17 (41.5%)	
Unknown	10	3 (30%)		7	5 (71.4%)		17	8 (47.1%)	

**Table 5:** Associations between DSG2 and clinical data in patients with EJA

Variable	*n*	Positive	%	Χ^2^	*P*
Patient age (years)					
<60	19	5	26.3%	0.093	0.76
≥60	77	23	29.9%		
Sex					
Male	77	24	31.2%	0.755	0.385
Female	19	4	21.1%		
Tobacco use					
Yes	27	7	25.9%	0.978	0.613
No	65	19	29.2%		
Unknown	4	2	50.0%		
Alcohol use					
Yes	10	3	30.0%	0.96	0.619
No	38	9	23.7%		
Unknown	48	16	33.3%		
Depth of tumor invasion					
T1	3	0	0.0%	3.847	0.427
T2	5	3	60.0%		
T3	12	3	25.0%		
T4	48	15	31.3%		
Unknown	27	7	25.9%		
Lymph node metastasis					
N0	23	8	34.8%		
N1	13	5	38.5%	1.93	0.749
N2	14	4	28.6%		
N3	18	3	16.7%		
Unknown	28	8	28.6%		
TNM stage					
I	6	1	16.7%	2.706	0.608
II	8	4	50.0%		
III	45	12	26.7%		
IV	24	8	33.3%		
Unknown	13	3	23.1%		

## Discussion

The application of serum biomarkers in cancer screening, monitoring response to treatment, and diagnosis is increasingly being investigated by researchers, mainly due to their greater acceptance and wider accessibility [30]. In recent years, some tumor-specific proteins have been identified as biomarkers for various cancers to assist and guide final diagnosis; for example, cancer antigen 125 (CA125) for ovarian cancer [31], carbohydrate antigen 19-9 [32,33] for pancreatic cancer, α-fetoprotein (AFP) [34,35] for hepatocellular cancer, carcinoembryonic antigen (CEA) [36] for colorectal cancer, and prostate-specific antigen (PSA) [37] for prostate cancer. CEA, cytokeratin 19 fragment (CYFRA21-1), and squamous cell carcinoma antigen (SCCA) are the most commonly used biomarkers for detection of ESCC [38]; however, their accuracy is not ideal [39,40]. Therefore, identification of sensitive and specific serum or plasma biomarkers for non-invasive diagnosis of ESCC remains a clinical challenge. Our study demonstrates that DSG2 is a potential candidate biomarker for ESCC.

In the current study, we measured the expression levels of DSG2 in serum from patients with ESCC and EJA, and normalized the results using a standard reference, to minimize between-plate variation. Serum DSG2 levels in patients with ESCC and EJA were significantly higher than those in healthy controls (Figure 1), indicating that DSG2 is a potential serological markers for detection of ESCC and EJA. Our findings that DSG2 is up-regulated in ESCC and EJA are consistent with reports on other cancers. Proteomics analysis of tissues from patients with pancreatic ductal adenocarcinoma identified DSG2 as among the top four candidate up-regulated proteins, and serum validation showed significant elevation of DSG2 levels in cancer patient samples [41]. Moreover, patients with ovarian cancer with high serum levels of shed DSG2 had significantly shorter progression-free and overall survival than those with lower DSG2 levels [16]. Klessner et al. found that MMP and ADAM10 caused shedding of the extracellular domain of DSG2 [42,43], and shed DSG2 can be detected in the serum of xenograft tumor models [44]. In addition, cleaved DSG2 enhances the proliferation of intestinal epithelial cells by interacting with HER2 or HER3, to activate the Akt/mTOR and MAPK signaling pathways [45]. These results suggest that DSG2 or cleaved DSG2 may be involved in ESCC progression; however, the role and regulation of cleaved DSG2 fragments in ESCC cells require further investigation.

Additionally, in our current study, serum DSG2 levels were demonstrated to serve as a diagnostic marker for patients with early ESCC and EJA. Measurement of DSG2 exhibited an AUC value of 0.724 with a sensitivity of 38.1% and specificity of 84.8% for diagnosis of ESCC in the training cohort, and these data were further verified in the validation and joint cohorts. Regarding EJA, serum DSG2 expression levels showed an AUC value of 0.698, associated with 29.2% sensitivity and 90.2% specificity. Notably, similar results were obtained for early-stage ESCC and early-stage EJA in a relatively small number of cases. The value of our study assessing serum DSG2 for diagnosis of early ESCC and EJA would likely have been significantly improved by recruitment of a larger number of early-stage cases. In the joint ESCC cohort, serum DSG2 levels were analyzed in 19 patients with early ESCC, and the results showed that it achieved the AUC value of 0.725. These findings suggest that serum DSG2 may be a marker for the diagnosis of gastrointestinal cancer.

In the present study, although we demonstrated that DSG2 is a useful biomarker for the diagnosis of ESCC and EJA, its sensitivity requires improvement; however, to our knowledge, this is the first report on the diagnostic value of serum DSG2 for early-stage cancer. Furthermore, most ESCC and EJA patients are already in advanced stages, which makes it difficult to recruit more patients in the early stages. Further investigation with large sample size can be conducted in multiple institutions, which is helpful to better evaluate the diagnostic value of DSG2 as a biomarker. In addition, previous studies have shown that the combined detection of multiple serum proteins as a single panel can increase the sensitivity or specificity of a single biomarker [29,46,47]. Therefore, one of the limitations of our study was that DSG2 was not used in combination with common markers (such as CEA, CYFRA21-1, and SCCA) for the diagnosis of ESCC and EJA. The high sensitivity of DSG2 is expected to be useful for early diagnosis of ESCC and EJA and to improve the prognosis of patients with these diseases. We hope that DSG2 can serve as a complement to specific tumor-markers for the diagnosis of ESCC and EJA, such as CEA.

## Conclusion

Taken together, our data provide evidence that serum DSG2 is higher in patients with ESCC and EJA and that analysis of serum DSG2 concentration generates novel and useful information for diagnosis of ESCC and EJA; however, the sample size of patients with early-stage ESCC and EJA was relatively small in the present study. Further verification of the diagnostic value of DSG2 in a larger sample set is warranted.

## Data Availability

The patient serum sample data involved in the present paper have been integrated into the statistics presented in the paper. Since no other data were used, relevant links cannot be provided; however, if other information or data related to the present paper is required, the authors can be contacted directly.

## Abbreviations

ADAM: a disintegrin and metalloprotease
AJCC: American Joint Committee on Cancer
AUC: area under the ROC curve
CEA: carcinoembryonic antigen
CI: confidence interval
CYFRA21-1: cytokeratin 19 fragment
DSG2: desmoglein-2
EJA: esophagogastric junction adenocarcinoma
ELISA: enzyme-linked immunosorbent assay
ESCC: esophageal squamous cell carcinoma
MMP: matrix metalloprotease
OD: optical density
ROC: receiver operating characteristic
SCCA: squamous cell carcinoma antigen
TNM: tumor node metastases
